# Web-Based Skin Cancer Assessment and Classification Using Machine Learning and Mobile Computerized Adaptive Testing in a Rasch Model: Development Study

**DOI:** 10.2196/33006

**Published:** 2022-03-09

**Authors:** Ting-Ya Yang, Tsair-Wei Chien, Feng-Jie Lai

**Affiliations:** 1 Department of Family Medicine Chi Mei Medical Center Tainan Taiwan; 2 Department of Medical Research Chi-Mei Medical Center Tainan Taiwan; 3 Department of Dermatology Chi-Mei Medical Center Tainan Taiwan

**Keywords:** skin cancer assessment, computerized adaptive testing, naïve Bayes, k-nearest neighbors, logistic regression, Rasch partial credit model, receiver operating characteristic curve, mobile phone

## Abstract

**Background:**

Web-based computerized adaptive testing (CAT) implementation of the skin cancer (SC) risk scale could substantially reduce participant burden without compromising measurement precision. However, the CAT of SC classification has not been reported in academics thus far.

**Objective:**

We aim to build a CAT-based model using machine learning to develop an app for automatic classification of SC to help patients assess the risk at an early stage.

**Methods:**

We extracted data from a population-based Australian cohort study of SC risk (N=43,794) using the Rasch simulation scheme. All 30 feature items were calibrated using the Rasch partial credit model. A total of 1000 cases following a normal distribution (mean 0, SD 1) based on the item and threshold difficulties were simulated using three techniques of machine learning—naïve Bayes, k-nearest neighbors, and logistic regression—to compare the model accuracy in training and testing data sets with a proportion of 70:30, where the former was used to predict the latter. We calculated the sensitivity, specificity, receiver operating characteristic curve (area under the curve [AUC]), and CIs along with the accuracy and precision across the proposed models for comparison. An app that classifies the SC risk of the respondent was developed.

**Results:**

We observed that the 30-item k-nearest neighbors model yielded higher AUC values of 99% and 91% for the 700 training and 300 testing cases, respectively, than its 2 counterparts using the hold-out validation but had lower AUC values of 85% (95% CI 83%-87%) in the k-fold cross-validation and that an app that predicts SC classification for patients was successfully developed and demonstrated in this study.

**Conclusions:**

The 30-item SC prediction model, combined with the Rasch web-based CAT, is recommended for classifying SC in patients. An app we developed to help patients self-assess SC risk at an early stage is required for application in the future.

## Introduction

### Background

Skin cancer (SC) is the most common malignant neoplasm occurring in White populations, and it is mainly divided into (1) malignant melanoma (MM) and (2) nonmelanoma SCs (NMSCs), which include squamous cell carcinoma and basal cell carcinoma as the major subtypes. The global incidence of MM and NMSCs is well-established and on the rise. In Australia, SC accounts for most newly diagnosed cancers each year, with age-standardized incidence rates for MM of 65.3×10^−5^ and 1878×10^−5^ for NMSCs [1,2]. There are >434,000 people in a population of only 23 million who treat keratinocyte cancer each year in Australia [2], causing a substantial socioeconomic burden and impact on public health services.

There are several well-recognized risk factors that increase the potential for the development of SC and have been reported in previous literature, such as UV radiation, genetic susceptibility, smoking, ionizing radiation, and the use of photosensitizing drugs [3]. Among the aforementioned risk factors, excessive UV radiation exposure remains the major causative risk factor for SC [4]. Therefore, it is crucial to modify personal behaviors to reduce direct and excessive sun exposure, such as avoiding long-term sunbathing or the use of indoor tanning devices, appropriately applying sunscreens, using sun-protective cloth garments, and staying in the shade.

### Requirement for Prediction Model in Classification of SC

In practice, it is difficult to provide people with their individual risk of SC [1]. Given the lack of clear recommendations for organized SC screening, physical exploration, clinical history of lesion changes, and correlated family SC history continue to be key for detecting skin neoplasms. Assuming that a person has attributes that highly correlate with the underlying architecture of the skin, the potential risk of SC can be assessed through questions (ie, questionnaire items); for example, underlying pigmentation traits include hair color, eye color, the propensity to freckle and sunburn, skin phenotypes, and some personal behavior factors such as tanning attitudes and sunbed use. Accordingly, it is feasible to construct a unidimensional scale to measure these attributes using the responses to the unidimensional items and further calculate an overall SC risk score using an assessment tool (eg, web-based computerized adaptive testing [CAT] administrations [1]) or even classify the SC risk for patients in clinical settings.

### Predicting SC Risk and Classifying the SC Possibility

Statistical validity is based on the correlations among item measures (or scores) on a questionnaire and people’s unobservable true status (eg, melanoma status–deemed latent traits that cannot be directly detectable in the real world) [5]. The Rasch model [6] is a mathematical modeling approach that has been used to assess how well the items measure the underlying latent traits [7-13], which are based on a unidimensional scale when the data fit the Rasch model’s expectation (ie, all items can be added to a summation score) [10,13]. Nonetheless, no SC classifications that use machine learning to predict SC risk have been illustrated and demonstrated in the literature. We are motivated to develop a prediction model for classifying SC in adults who are potentially at risk.

### CAT Assessment and Limitation in SC Classification

CAT is a tailored measure based on item response theory (IRT) [14,15] that can better align with each examinee’s ability level [10,13,16]. The computer follows an IRT-based algorithm, and the difficulty of the next selected item depends mainly on all previously answered items. As such, each patient needs to answer the fewest possible items by dynamically selecting appropriate testing items, resulting in less respondent burden without compromising measurement precision and thereby making it possible to individualize each participant’s assessment [1,10,13].

The limitation of CAT applied to machine learning is the missing responses (ie, unanswered items) in the data. Fortunately, generating the expected responses to endorse the answers in CAT has been resolved to overcome the drawback of not having all the items answered in CAT (ie, using the expected value to fill in the missing data, as done in previous studies [13,17,18]). As such, convolutional neural networks (CNNs) [19,20] combined with the expected responses to classify the groups of individual bullying levels [13] are applicable. Thus, we are interested in applying the expected responses to CAT to (1) reduce participant burden with more accurate outcomes [1,10,13,16] and (2) predict SC classification in patients.

### Web-Based Assessment Using Smartphones

With the advent of the era of digital technology, the advancement and maturation of mobile health and health communication technology have been rapidly increasing [21]. To date, smartphone apps for classifying SC using CAT-based machine learning for patients in health care settings are lacking when searching for publications in the PubMed library using the keywords *skin and cancer* AND *computerized adaptive testing* AND *CAT* AND *machine learning* as of December 5, 2021. It is not only the complexity of the CAT procedure with multimedia illustrations embedded into a web-based module but also the difficulty of the model’s parameters that need to be transformed into the probability of classification types when SC is assessed on the web. A web-based CAT app incorporating machine learning and SC could provide patients with a better understanding of the SC classification and prediction of SC at risk before a serious SC problem occurs.

### Study Aims

The aims of this study are to (1) compare the prediction accuracy of SC between machine learning models in SC classification and (2) build a CAT-based SC assessment using machine learning to develop an app for automatic classification of SC to help patients assess SC risk at an early stage.

## Methods

### Data Source

On the basis of a previous study [1,22], we extracted data from a population-based Australian cohort study of SC risk (N=43,794) by simulating Rasch data [23], including 1000 virtual patients across 30 feature variables defined in the previous study [1] (Multimedia Appendix 1).

All data used in this study were simulated and extracted from the previous article [1]. Given that this study design uses simulation data, ethical approval was not required according to the Taiwan Ministry of Health and Welfare regulations.

### Characteristics of the Simulated Data

#### The Original Survey Data

The original data were retrieved from the baseline questionnaire in the QSkin Sun and Health study [22]. A population-based cohort study of 43,794 men and women aged 40 to 69 years was randomly sampled from the population of Queensland, Australia [1], to obtain a calibration data set (two-thirds; 29,314/43,794, 66.94%) and a validation data set (one-third; 14,480/43,794, 33.06%). In the calibration data set, 24.61% (7213/29,314) of participants had a history of SC, and 75.39% (22,101/29,314) of participants did not.

#### The Study Simulation Data

For simplification, the 30-item difficulties calibrated in the previous study [1] (Table 1) using the Rasch partial credit model [24] were applied to yield 1000 virtual cases following a normal distribution (mean 0, SD 1; see the demonstration in Multimedia Appendix 1 with an MP4 video). The suggested cutoff point was set at 0.88 logits [1] to determine the 2 groups of cancer and noncancer in the simulation data. As such, the data with 1000 people × 30 items and 1 label (ie, 1 and 0 for melanoma status defined as cancer and noncancer groups) were applied in this study with the following 2 sections (ie, 3 models and 3 tasks).

**Table 1:** Overall and threshold difficulties in logit (log odds) across the 30 items.

Number	Variable	Overall difficulty	Threshold difficulty			
			Step 1	Step 2	Step 3	Step 4
1	Gender (male as 1 and female as 0)	0.16	0.00	N/A^a^	N/A	N/A
2	Skin color on areas never exposed to the sun?	−2.32	−2.46	0.78	1.68	N/A
3	Your behavior in the strong sun for 30 minutes at noon?	−0.17	−1.51	0.41	1.10	N/A
4	Your behavior outdoors in the sun without protecting your skin?	−0.49	−0.85	−0.42	1.27	N/A
5	What color are your eyes?	−0.11	−0.04	0.60	1.55	−2.11
6	What was your natural hair color at the age of 21 years?	0.48	0.70	−0.83	−1.30	1.43
7	How many freckles were on your face at the age of 21 years?	0.72	−0.37	0.01	0.36	N/A
8	How many moles did you have on your skin at the age of 21 years?	0.76	−1.45	0.53	0.92	N/A
9	How many times in your whole life have you used sunbeds?	1.27	1.35	0.30	−0.75	−0.69
10	How many separate skin cancers have you ever had excised from your skin?	0.98	0.45	−1.36	1.30	−0.39
11	How many separate sunspots or skin cancers have you ever had frozen or burnt off on your skin?	0.53	−0.05	0.49	−0.22	−0.11
12	Have I been told that I have melanoma?	0.99	0.99	N/A	N/A	N/A
13	Will you get melanoma at some point in the future?	0.26	−1.14	−0.82	1.14	0.82
14	How many times were you sunburned so badly that you were sore for at least 2 days or your skin peeled as a child?	0.58	−1.41	0.37	0.11	0.36
15	How many times were you sunburned so badly that you were sore for at least 2 days or your skin peeled in your teenage years?	0.17	−2.40	0.35	0.27	0.74
16	How many times were you sunburned so badly that you were sore for at least 2 days or your skin peeled in adulthood?	0.58	−1.83	0.59	0.10	0.46
17	How many hours did you spend outdoors and in the sun from Monday to Friday in the past year?	0.29	−0.04	0.44	−0.39	N/A
18	How many hours did you spend outdoors and in the sun from Monday to Friday at the age of 10 to 19 years?	−0.51	−0.65	0.24	0.41	N/A
19	How many hours did you spend outdoors and in the sun from Monday to Friday at the age of 20 to 29 years?	−0.15	−0.46	0.41	0.05	N/A
20	How many hours did you spend outdoors and in the sun from Monday to Friday at the age of 30 to 39 years?	0.04	−0.29	0.42	−0.13	N/A
21	How many hours did you spend outdoors and in the sun during Saturday and Sunday in the past year?	−0.14	−0.42	0.23	0.19	N/A
22	How many hours did you spend outdoors and in the sun during Saturday and Sunday at the age of 10 to 19 years?	−0.94	−0.46	0.21	0.26	N/A
23	How many hours did you spend outdoors and in the sun during Saturday and Sunday at the age of 20 to 29 years?	−0.72	−0.60	0.18	0.43	N/A
24	How many hours did you spend outdoors and in the sun during Saturday and Sunday at the age of 30 to 39 years?	−0.45	−0.56	0.19	0.37	N/A
25	Routinely apply sunscreen to my face	−0.46	0.00	N/A	N/A	N/A
26	Routinely apply sunscreen to my hands and forearms	−1.80	0.00	N/A	N/A	N/A
27	Routinely apply sunscreen to other parts of my body	−2.56	0.00	N/A	N/A	N/A
28	Routinely apply sunscreen going out in the sun: no	−0.36	0.00	N/A	N/A	N/A
29	Whether applying sunscreen outside in the sun?	−0.31	−0.90	−0.16	1.06	N/A
30	How often have you been outside in the sun in the past year?	0.45	−0.77	−0.08	0.85	N/A
31	Melanoma status (label as cancer and noncancer group)	N/A	N/A	N/A	N/A	N/A

^a^N/A: not applicable.

### The 3 Models of Machine Learning Used in Microsoft Excel

#### The 3 Models Applied in This Study

Three models of machine learning—naïve Bayes (NB) [25], k-nearest neighbors (KNN) [26], and logistic regression (LR) [27-31]—were applied to compare the model accuracy of classifying SC in the 1000×30 rectangle data set. The 2 training (70%) and testing (30%) sets (ie, the hold-out validation) were separated to examine the model’s accuracy with a proportion of 70:30, where the former was used to predict the latter.

We calculated the sensitivity, specificity, receiver operating characteristic curve (area under the curve [AUC]), and CIs along with the accuracy and precision across the 3 aforementioned models for comparison. In addition, k-fold cross-validation was performed for the 3 models using the Weka software (University of Waikato) [32]. If the Weka Explorer (graphical user interface) and the *Classify* tab are selected, we can find it by looking for the *Choose* button under the *Classify* tab. Once we navigate through the folders, the 3 classifiers are used (ie, NB classifiers→Bayes→NB; instance-based learner [IBk] classifiers→lazy→IBk; and classifiers→functions→logistic). For instance, once we select IBk for the KNN classifier, we click on the box immediately to the right of the button. This will open up a large number of options. If we then click on the button *More* in the *Options* window, we will see all the options explained. We can do this for all the classifiers to obtain additional information (eg, NB, logistic, or more; see the demonstration using an MP4 video in Multimedia Appendix 2). Meanwhile, more information about the 3 models is provided in Multimedia Appendix 3.

#### Calculation of Model Accuracy

After the parameters in the selected model are estimated, the accuracy of a model in the training and testing sets can be obtained through the following equations [33,34]:

The accuracy was determined by observing the higher sensitivity, specificity, precision, accuracy, and AUC in the models. The definitions are as follows:

True positive (TP) = the number of predicted cancers to the true SCs **(1)**

True negative (TN) = the number of predicted non-SCs to the true noncancers **(2)**

False positive (FP) = the number of noncancers – the number of TN **(3)**

False negative (FN) = the number of cancers – the number of TP **(4)**

Sensitivity = TP rate = TP/(TP + FN) **(5)**

Specificity = TN rate = TN/(TN + FP) **(6)**

Precision = positive predictive value = TP/(TP + FP) **(7)**

Accuracy = (TP + TN)/N **(8)**

N = TP + TN + FP + FN **(9)**

AUC = (1 − specificity) × sensitivity/2 + (sensitivity + 1) × specificity/2 **(10)**

SE for AUC = √(AUC × [1 – AUC]/N) **(11)**

95% CI = AUC ± 1.96 × SE for AUC **(12)**

Similarly, the confusion matrix can be made when the true conditions (ie, SC and non-SC) and the predictions (ie, positive and negative) are known in the predicted training set (or the testing data set) matched to the label (ie, 1 and 0 as cancer and noncancer groups) in the training set. Other indicators in equations (1) to (12) can be obtained accordingly.

It is worth noting that we made the model residual with the average values in the 2 groups (ie, average [range in the group of SC] + average [range in the group of non-SC]) to overcome the imbalance class data. As such, the AUC for sensitivity and specificity could be balanced in reports [35]. Details about the setting formula are provided in the Microsoft Excel module in Multimedia Appendix 1.

### The 3 Tasks

#### Feature Variables Shown on a Forest Plot (Task 1)

The 30 variables [1] were shown on a forest plot [36-38] via the following steps: standardize each variable based on the mean (0) and SD (1) and compare the standardized mean difference on a forest plot [39].

The chi-square test was conducted to evaluate the heterogeneity between variables. Forest plots (CI plots) were drawn to display the effect estimates and their CIs for each indicator.

#### Comparing the Accuracies in Models (Task 2)

We calculated the sensitivity, specificity, AUC, and CIs along with the accuracy and precision across the proposed models in comparison using equations (1) to (12). Both AUCs in the training and testing sets were compared to assess the model accuracy and stability [34,35].

#### SC Risk and Classification (Task 3)

##### The Rasch Model and the First-Order Derivative in Calculus

In the Rasch model, the probability can be expressed as follows:



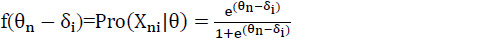

**(13)**


where *θ* is the person’s ability, and *δ* is the item difficulty for person *n* and item *i*, respectively. The processes of the first-order derivative on *θ* are described below:



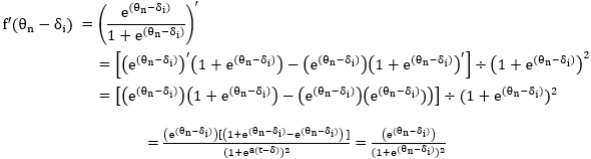

**(14)**


##### The Newton-Raphson Iteration Method

The Newton-Raphson iteration method, one of the essential iteration techniques for parameter estimation, has been frequently mentioned in the methodology literature [40-43] and popularly used in practice with the Rasch model [44,45].

A revised estimated measure, *θ*_m_ + 1, is obtained from the previous measure of *θ*_m_ and the adjustment by the residual and the summed variance (defined by f'[*θ*_m_ – *δ*_i_] across all answered items in equation 15):



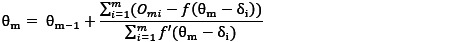

**(15)**


The CAT SE is defined by the following equation:



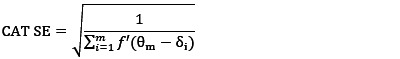

**(16)**


The next selected item is determined by the maximum information (variance = f'[*θ*_m_ – *δ*_i_]) of the item in all answered items shown in the following equation:

Information_i_ = f'(*θ*_m_ – *δ*_i_) **(17)**

##### CAT Stop Criterion

The CAT termination is set at the CAT SE smaller than the SE of measurement (SEM) [1,46].

SEM = SD √(1 – *Rel*) **(18)**

*Rel* is the Cronbach α of the questionnaire. Therefore, if there is a test (or questionnaire) with an SD of 1.0 logits and a Cronbach α of .78 [1], the SEM would be 0.469 (1 × √[1 – .78]).

**Figure 1:**
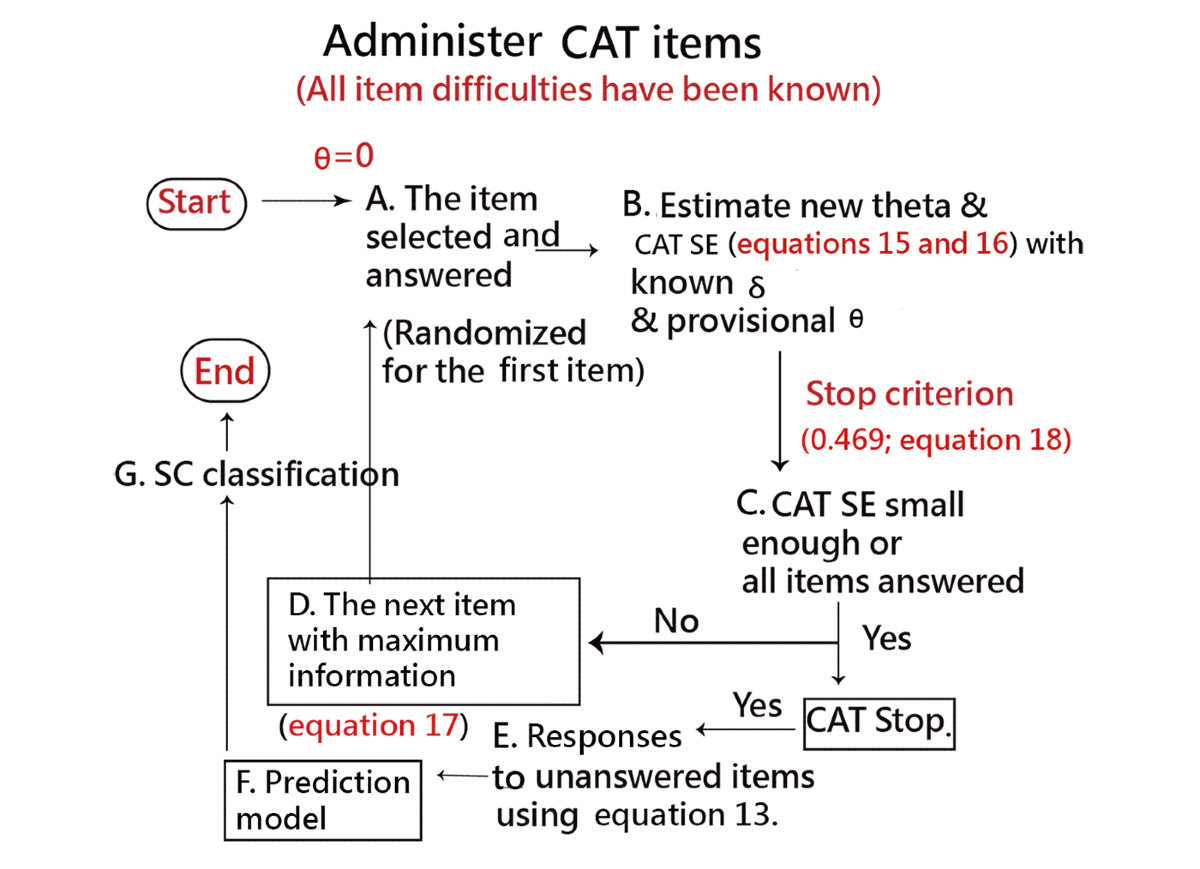
SC–CAT process and SC classification using machine learning. CAT: computerized adaptive testing; SC: skin cancer.

If CAT is terminated, the responses to unanswered items are filled in with their expected values using equation (13) when the final measure is known. The SC classification is then performed (Figure 1).

##### The Fit Statistics of the Mean Square Error

The Rasch fit statistics of mean square errors (MNSQs), including infit and outfit [40,41], are shown on the SC CAT to represent the extent of the deviation from the expectation of the Rasch model for the examinee’s responses.



Infit MNSQ =

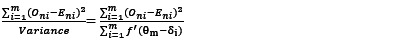
**(19)**


Outfit MNSQ = 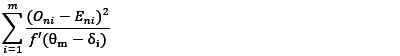

**(20)**
where *O_ni_* is the observed response for person *n* on item *i*, and *E_ni_* is the corresponding expected value in equation (13). The variance is referred to in equations (14) and (17).

Again, another way to judge a person’s responses depends on the *Z* score (denoted by *Z*) in equation (21). According to the Rasch model, these accumulated *Z*^2^ values ought to follow a chi-square distribution with 1 degree of freedom (denoted by *df*) for each *Z*^2^ value minus the degree of freedom necessary to estimate the person measure *θ*_n_ [47]. Any sum of *Z*^2^, when divided by its *df*, should follow the mean square distribution in equation (22). This can conveniently be evaluated as the *t* statistic, which has approximately a unit normal distribution (ie, *N*[0,1]) [46], shown in equation (23).



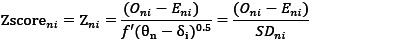

**(21)**




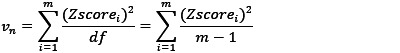

**(22)**




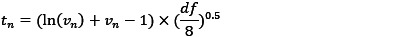

**(23)**


##### The Skin Cancer–Computerized Adaptive Testing Algorithm

Wright [48] suggests a simpler algorithm for classroom use, classification, and performance tracking in a low-stakes environment. This algorithm is easy to implement and could be successfully used at the end of each learning module to keep track of the persons’ responses in the process [46]. Figure 1 shows the core steps of skin cancer–computerized adaptive testing (SC–CAT) needed for practical adaptive testing using the Rasch model:

1. Start with a patient at an initial θ (SC score in logit) of 0.
2. Find a randomized item from the item poll via the SC–CAT.
3. Respond to the item with difficulty and the corresponding threshold δ (difficulty; label A in Figure 1).
4. Calculate the provisional θ in equation (15) based on the known item difficulties (label B).
5. Examine whether the CAT stop criterion (ie, SEM=0.469) is reached in equations (16) and (18) (label C).
6. Select the next item in equation (17) if the SC–CAT continues (label D).
7. Return to Step 3.
8. Fill in the expected values of the unanswered items via equation (13) when the SC–CAT stops based on the final estimated θ (label E).
9. Perform the prediction model (label F).
10. Obtain the classification (ie, SC or non-SC; label G).

#### The App Developed in This Study

An app for the detection of SC in adults was designed and developed. A 30-item self-assessment app using mobile phones was designed to predict and classify SC using machine learning and model parameters. The model parameters were embedded in the computer module.

The results of the classification (ie, SC+ and SC–) instantly appear on smartphones. A visual representation displaying the classification effect is plotted using 2 curves (ie, one from the bottom left to the top right corner denotes the success [SC+] feature, and another from the top left to the bottom right is the failure [SC–] attribute). The visual dashboard with binary (ie, SC+ and SC–) category curves is shown on Google Maps.

### Statistical Tools and Data Analysis

MedCalc 9.5.0.0 for Windows (MedCalc Software) was used to calculate the sensitivity, specificity, and the corresponding AUC using LR when the observed labels (ie, 0 for SC– and 1 for SC+) and the predicted probabilities (ie, the continuous variable in equation 13) were applied.

Author-made modules in Microsoft Excel were applied to compute the model prediction indicators expressed in equations (1) to (12). The three proposed models—NB, KNN, and LR—were performed using Microsoft Excel and Weka [32] (Multimedia Appendix 1 and 2). The web-based CAT was programmed using the classic active server pages.

**Figure 2:**
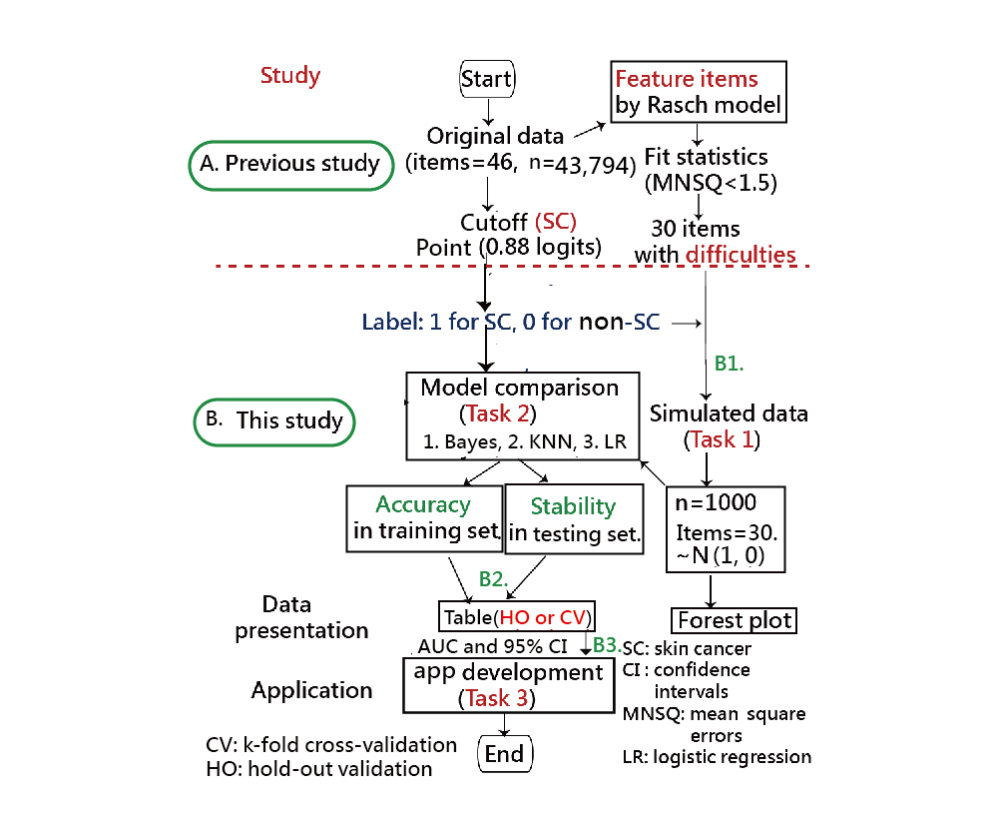
Two major parts are in the study flowchart (in the upper and bottom panels), and three tasks are in the bottom panel. AUC: area under the curve; KNN: k-nearest neighbors; MNSQ: mean square error; SC: skin cancer; HO: hold out validation.

The study flowchart (shown in Figure 2) comprises two parts: one is from the previous study [1] and another includes 3 models. A total of 3 tasks are elaborated in this study. The abstract video is provided in Multimedia Appendix 1 as well.

### Ethics Approval and Consent to Participate

Not applicable. All data were simulated and extracted from a previous study [1].

### Availability of Data and Materials

All data used in this study are available in the Multimedia Appendices.

## Results

### Task 1: Feature Variables Demonstrated on a Forest Plot

**Figure 3:**
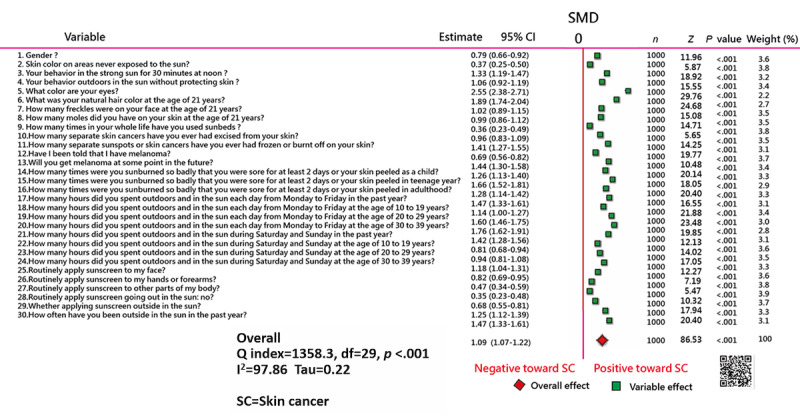
Using the forest plot to display feature variables on smartphones [49] or clicking the QR Code. SMD: standardized mean difference.

The 30 variables are presented in a forest plot (Figure 3). We can see that all green boxes are on the right side beyond the mean standardized mean difference (0), indicating that the variables are eligible (*P*<.05) for discriminating the melanoma status (ie, SC and non-SC groups).

### Task 2: Comparing the Accuracies Between Models

A comparison of the model accuracies is shown in Table 2. We can see that all AUCs are >0.80 in models across the training and testing sets. The 30-item KNN model yielded higher AUC values of 99% and 91% for the 700 training and 300 testing cases, respectively, far beyond the other 2 models (ie, NB and LR; Table 3). However, if k-fold cross-validation is performed, the 30-item KNN model yields lower AUC values of 85% (95% CI 83%-87%), shown in Table 4.

**Table 2:** Comparison of model accuracy and stability using simulation data (hold-out validation).

Study model	Training cases/testing cases, N	Accuracy ≥0.80 (training sets)						Stability ≥0.70 (testing sets)				
		Sensitivity	Specificity	Precision	Accuracy	AUC^a^	Sensitivity		Specificity	Precision	Accuracy	AUC
Naïve Bayes	700/300	0.92	0.89	0.82	0.90	0.90	0.79		0.98	0.97	0.91	0.89
KNN^b^	700/300	0.98	0.99	0.98	0.99	0.99	0.83		0.99	0.99	0.93	0.91
LR^c^	700/300	0.82	0.91	0.84	0.88	0.87	0.70		0.92	0.85	0.84	0.81

^a^AUC: area under the curve.

^b^KNN: k-nearest neighbors.

^c^LR: logistic regression.

**Table 3:** Comparison of model accuracy and stability using simulation data (95% CIs of the area under the curve [AUC] for hold-out validation)^a^.

Study model	Accuracy ≥0.80 (training sets)				Stability ≥0.70 (testing sets)		
	Training cases, N	AUC (95% CI)	Significant difference	Testing cases, N		AUC (95% CI)	Significant difference
Naïve Bayes (1)	700	0.90 (0.88-0.92)	1, 2	300		0.89 (0.85-0.93)	—^b^
KNN^c^ (2)	700	0.99 (0.98-1.00)	1, 3	300		0.91 (0.88-0.94)	3
LR^d^ (3)	700	0.87 (0.85-0.89)	1, 2	300		0.81 (0.77-0.85)	2

^a^The computation of the 95% CI for the AUC is referred to in equations (10) to (12).

^b^Data not available.

^c^KNN: k-nearest neighbors.

^d^LR: logistic regression.

**Table 4:** Comparison of model accuracy and stability using simulation data (k-fold cross-validation).

Study model	Training cases/testing cases, N	Accuracy ≥0.80 (training sets)						Stability ≥0.70 (testing sets)	
		Sensitivity	Specificity	Precision	Accuracy	AUC^a^	AUC (95% CI)		Significant difference
Naïve Bayes (1)	700/300	0.93	0.92	0.87	92.40	0.98	0.98 (0.97-0.99)		2
KNN^b^ (2)	700/300	0.87	0.90	0.84	89.20	0.85	0.85 (0.83-0.87)		1, 2
LR^c^ (3)	700/300	0.90	0.90	0.90	92.40	0.98	0.98 (0.97-0.98)		2

^a^AUC: area under the curve.

^b^KNN: k-nearest neighbors.

^c^LR: logistic regression.

### Task 3: Developing an App for SC Classification

**Figure 4:**
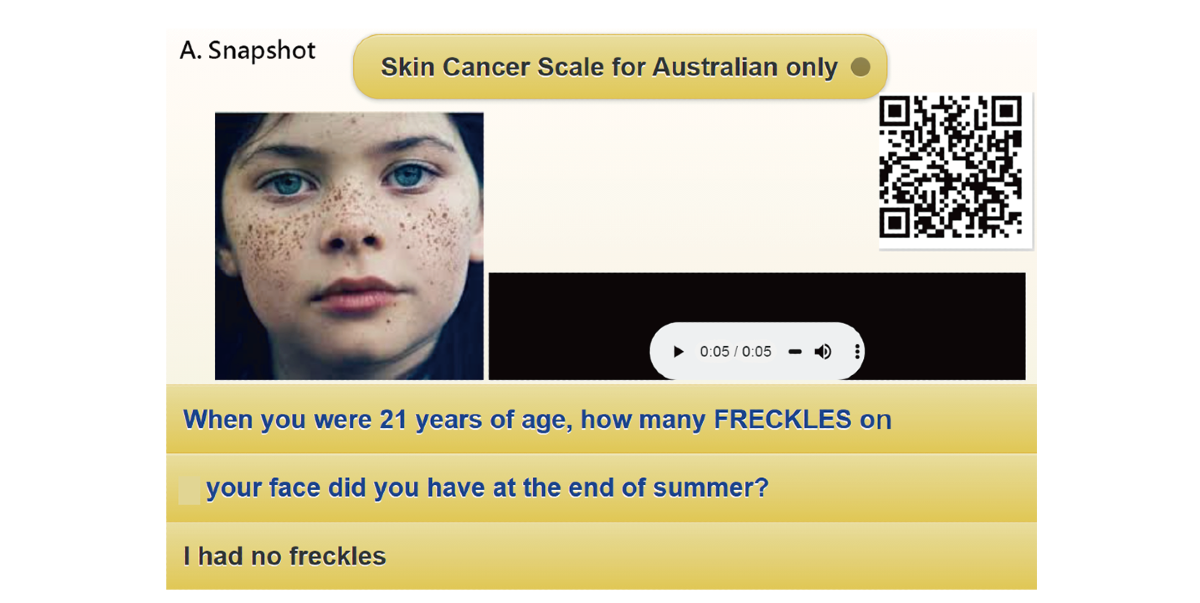
Snapshot of skin cancer assessment on smartphones from the web-based CAT model [50] or clicking the QR Code.

**Figure 5:**
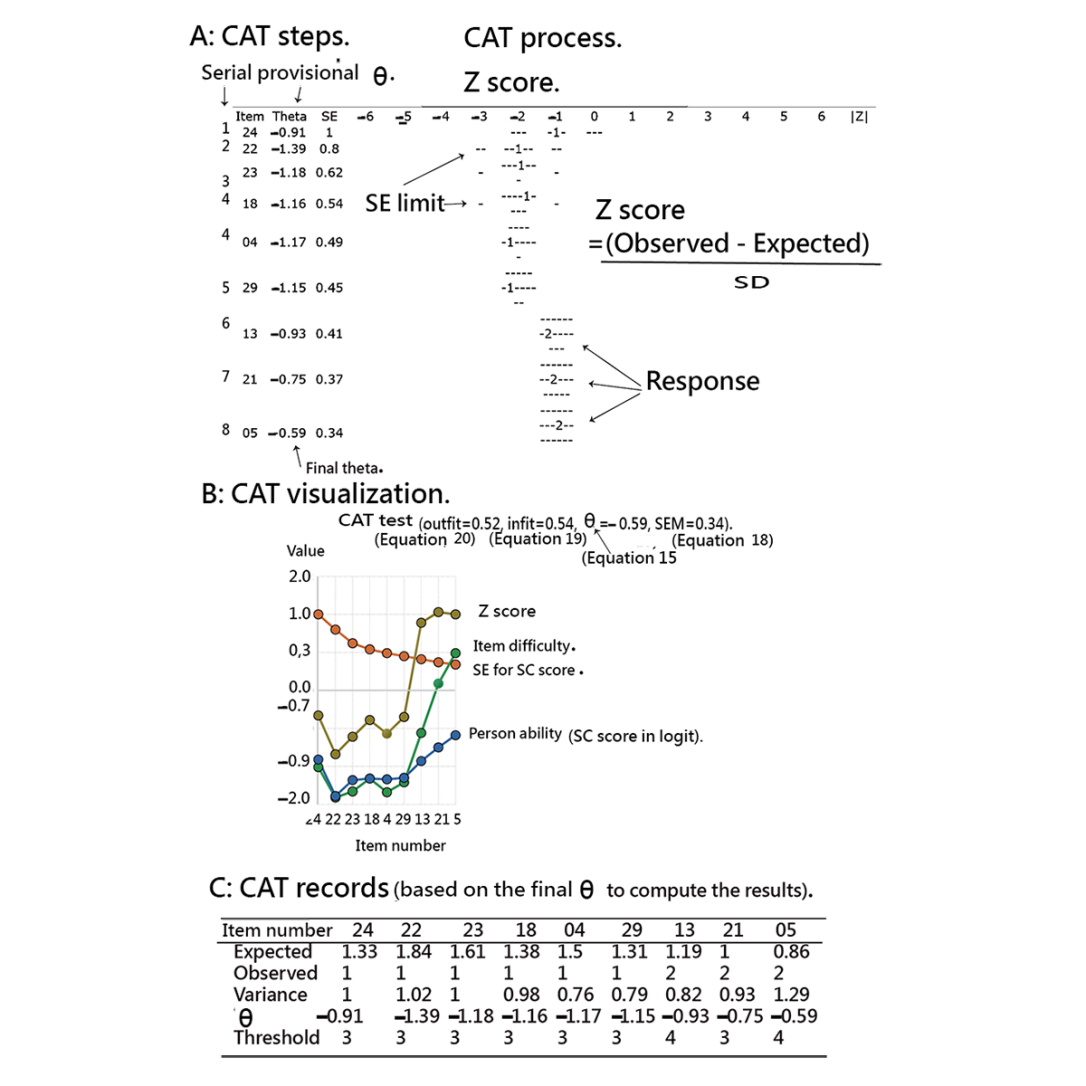
The process in SC-CAT on smartphones with three panels A, B, C denoted by steps, visualizations and records, respectively. CAT: computerized adaptive testing; SC: skin cancer; SEM: standard error of measurement.

**Figure 6:**
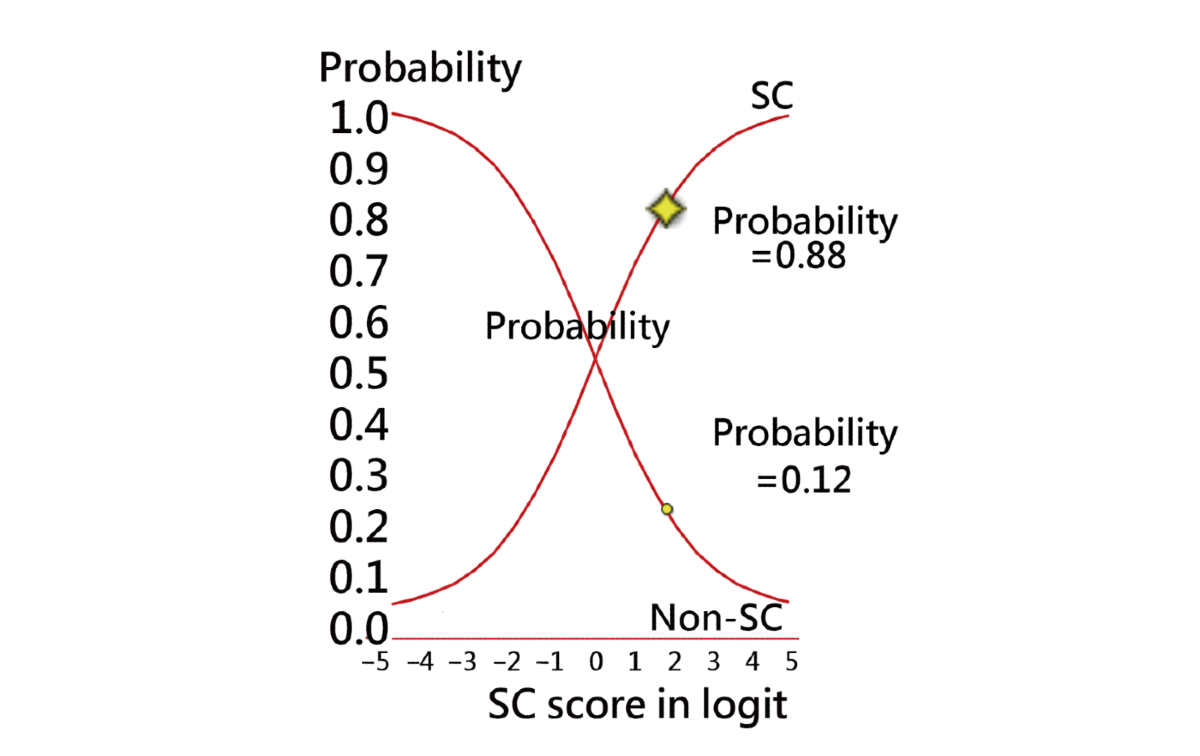
The result of SC+ assessment with classification and probability on smartphones. SC: skin cancer.

A screenshot obtained from a mobile phone used to respond to the questions is shown in Figure 4, the CAT process is shown in Figure 5, and the assessment results are shown in Figure 6. In this example, we can see that the item-by-item CAT process is displayed in Figure 5, and the patient has a high probability (0.88) of developing SC, as shown in Figure 6.

Readers are invited to scan the QR code in Figure 4 and practice the web-based CAT on their own. The CAT process is shown in Figure 5. The assessment of the calibration plot is shown in Figure 6.

We developed the CAT-based app for classifying SC in adults. The CAT process was demonstrated item by item and is shown in the 3 panels of Figure 5. Person *θ* is the provisional ability (eg, the third column in the top panel of Figure 5 or the blue line in the middle panel of Figure 5) estimated by the CAT module (equation 15).

The SEs (equation 16) are along the orange line in the middle panel of Figure 5 (or the dotted lines in the top panel of Figure 5). We can see that the more items responded to by a patient, the smaller the SEs will be. The SE was generated by the formula 1/√(Σinformation[*i*]) (equation 17), where *i* refers to the CAT items responded to by a patient.

In addition, the item difficulties (shown in Table 1) are along the green line in the middle panel of Figure 5. The residual is derived from the difference (observed – expected; bottom panel of Figure 5). The *Z* score (*i*) along the brown line is computed using equation (21), which equals the squared variance (*i*) shown in the bottom panel of Figure 5.

CAT will stop if the residual value is <0.05. The correlation coefficient between the CAT estimated measures and the step series numbers using the last 5 estimated *θ* values was computed. A flatter *θ* trend indicates a higher probability of a person’s measure converging to the final estimation.

It is worth noting that a person’s MNSQs (ie, infit and outfit at the top of the middle panel in Figure 5) are generated by the formula in equations (19) and (20). If the value of the outfit is >2.0 [51], the person’s response pattern is significantly aberrant beyond the model’s expectation. In the example shown in the middle panel of Figure 5, we can see that the patient’s response pattern with outfit MNSQ (0.52, less than the cutoff point of 2.0) and the *t* statistic (−0.95 = [ln(0.585) + 0.585 − 1] × 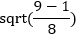, where *v* = 0.52 × 9/[9 – 1] based on equations (22) and (23) meets the expectation of the Rasch model rather well.

Once the CAT terminates, the resulting example is shown in Figure 6. We can see that the SC+ with a high probability (0.88) is shown on the curve of success from the bottom left to the top right corner. The sum of both probabilities (ie, SC+ and SC–) equals 1.0. The odds can be computed by the formula *p*/(1 – *p*) = 0.88/0.12 = 7.33, indicating that the patient had an extremely high probability or tendency toward SC+. It is worth noting that CAT substantially reduces participant burden (ie, only 9 items were responded to in the CAT, and 70% [(30 – 9)/30] efficiency gains were from the CAT) without compromising measurement precision.

### Web-Based Dashboards Shown on Google Maps

A total of 2 QR codes shown in Figures 3 and 4 (or links [49,50]) are provided for readers who can manipulate the dashboards on their own. In Figures 3 and 4, the animation-type dashboards make the data (eg, feature variables) and the app easier and clearer to understand once the QR Codes are clicked on.

## Discussion

### Principal Findings

We built a CAT-based model via a machine learning approach to develop an app to predict the classification of SC and help patients identify SC risk earlier to reduce participant burden and maintain acceptable measurement precision. A total of 1000 cases were simulated based on the item difficulties with a cutoff point of 0.88 logits to determine 2 groups (cancer and noncancer) using Rasch analysis addressed in a previous study [1]. A total of 3 types of machine learning (NB, KNN, and LR) were applied to compare the accuracy and stability of the models in SC classification. We observed that (1) the 30-item KNN model yielded higher AUC values of 99% and 91% for the 700 training and 300 testing cases, respectively, than its 2 counterparts using the hold-out validation but had lower AUC values of 85% (95% CI 83%-87%) in the k-fold cross-validation and (2) an app for patients that predicts SC classification was successfully developed and demonstrated in this study.

### Previous Research Using Computers to Diagnose SC Instead of Classifying SC

Melanoma is considered one of the fastest-growing and most aggressive SCs; it was first described as a “fatal black tumor” by Hippocrates in 5000 BC and was later recognized to have the propensity to metastasize by William Norris in 1820 [52]. It causes most of the deaths from SC. Therefore, timely and accurate recognition of melanoma combined with appropriate treatment regimens could optimize clinical outcomes and avoid potentially fatal metastasis. Although computer-based algorithms have been proposed to develop novel predictors of prognosis and improve the efficiency and diagnostic accuracy of cancer metastasis, significant challenges for SC prediction and classification still remain [52].

For instance, a report that sniffer dogs are able to detect MM at a curable stage was first described in the United Kingdom by William et al [53]. Thereafter, studies focusing on the utility of dog olfaction for screening or diagnosing different medical conditions, such as COVID-19, malignancies, diabetes, Parkinson disease, seizures, certain hormonal and enzymatic defects [54-67], and melanoma [53], ensued. Machine learning models based on CNNs were applied to extract the region of interest of the skin lesion data set and showed that training CNN models with the region of interest–extracted data set could improve the accuracy of the prediction [55-57].

A mobile CAT was developed to help people efficiently assess their SC risk [1]. However, no such classification of SC using machine learning was provided to readers before, as we did in Figure 4 of this study. This mobile assessment could be used to quickly estimate a person’s SC risk and educate patients about the need to implement skin protection and promote self-examination of the skin [68-70]. In particular, patients with a history of SC had a higher mean score of responses than those without a history of SC [1].

### Animation-Type CAT Module to Increase Health Literacy for Patients

Patients’ health literacy (eg, understanding their own SC risk) is increasingly considered a critical factor affecting patient-physician communication and health outcomes [71]. Populations with below-basic or basic health literacy are less likely to obtain health issue–related information from traditional printed sources such as newspapers, magazines, books, or brochures than those with higher health literacy [72]. A brief CAT, such as the one we developed in this study, could be used to inform people quickly about their potential risk of SC and help these individuals engage in sun-protective behaviors.

This CAT module is a practical tool that can efficiently identify suitable item subsets for each individual and, therefore, maximize the efficiency and precision of the entire testing process. Through CAT, it was found that it can save up to 42% (or more) of test length and achieve a very similar degree of measurement precision as a non-CAT. This is consistent with the literature [73-76].

The tool offers diagnostics that can help practitioners assess whether responses are distorted or abnormal. For example, outfit mean square values of ≥2.0 suggest an unusual response [51]. If responses do not fit well with the model’s requirement, they can be highlighted for suspected cheating, careless responding, lucky guessing, creative responding, or random responding [74]. Otherwise, one can take follow-up action (eg, medical consultation) to recheck the reasons for unexpected responses to questions [8,77,78] if the result shows a high cancer risk. Readers are invited to run the SC–CAT mobile app through the QR code, as shown in Figure 4.

### Strengths and Features of This Study

There are two major forms of standardized assessments in clinical settings [79]: (1) a traditional self-administered questionnaire and (2) a rapid short-form scale [80]. Each has its own advantages and shortcomings. Traditional pencil-and-paper questionnaires require higher financial investment and have a substantial burden on respondents resulting from the following rationale: participants need to answer questions that do not provide additional information about their personal risk of certain diseases to achieve adequate precision measurement [20]. In contrast, by administering items that are most informative for the examinee, the CAT can provide precise measurement of an examinee’s proficiency with the fewest possible items and then terminate at an appropriate number of items according to the required person reliability [1] (equation 18).

Second, not all questions were answered in the CAT. In contrast to those using the mean value [20] over the entire data set to fill in the missing values, we applied the expected value in the model for each unanswered question to fill in the missing data, as done in previous studies [13,24,25]. By doing so, the expected responses and model parameters can be applied to classify the SC groups. To date, we have not seen anyone using CAT combined with machine learning to classify SC in the literature, which is a breakthrough and the second feature of this study.

Third, as with all forms of web-based technology, advances in mobile health and health communication technology are rapidly emerging [21]. The use of mobile web-based CAT is promising and worth implementing in many fields for the assessment of health issues. The CAT graphical representations shown in Figure 4 are modern and innovative in academics.

Few studies have used machine learning to perform NB, KNN, and LR on Microsoft Excel, as we did in this study. These modules are provided in Multimedia Appendix 1, which is the fourth feature of this study.

We applied the LN algorithm along with the model’s parameters to design a routine on an app that is used to classify individual SCs (Figure 6), which is the fifth feature of this study. We have not seen any such SC–CAT combined with LN implemented on mobile phones before.

Different results were found when comparing the model accuracy of the AUC between the hold-out validation and the k-fold cross-validation (Tables 2, 3, and 4), which might be attributed to the small sample size (eg, 1000) used in this study. The evidence providing the k-fold cross-validation to improve the strength and confidence in the models’ evaluation is the sixth feature of this study.

### Limitations and Future Studies

Our study has some limitations. First, although the psychometric properties of the 30-item SC assessment have been validated for measuring SC risk [1], there is no evidence to support that the 30-item SC assessment is suitable for users outside of Australia. We recommend additional studies using their own database of SC assessment to estimate the item parameters and see whether a difference exists.

Second, although the Bayesian model performed better than the other 2 models (KNN and LR), CAT was incorporated with LR instead of the Bayesian model. The reason for this is that LR requires less computation time than the Bayes and KNN algorithms, as the latter uses pair-to-pair comparison in the algorithm. Future studies are encouraged to compare the efficiency and time consumption in computation between different models.

Third, the study was based on an article [1] that used the 30-item SC–CAT module. All the model parameters (ie, item difficulties and step-threshold difficulties) were derived from this study [1]. If any environment or condition is changed (eg, other populations in the country and different ethnicities), the result (eg, the model’s parameters) will be different from that of this study. The ethnicity of the study population was also a limitation. It is worth further verifying and investigating different populations and ethnic groups under the concept we used in this study.

Fourth, the SC assessment is a 1-dimensional construct. The item difficulties used to estimate a person’s measure were calibrated using Rasch Winsteps software. Traditionally, a person’s ability (*θ*) should be estimated by the CAT method, as previous studies have done [1,10,13,16]. In this study, the SC group should be further classified (eg, transforming the log odds to probability in LR and determining the SC group by observing the probability greater or less than 0.5). Different models applied to CAT will use disparate classification schemes. Future studies should be cautious on this matter.

Fifth, readers are encouraged to access the app by scanning the QR code in Figure 4. Professional practical apps should be further developed for Android and iOS systems in the future.

Finally, the study sample was retrieved from the baseline questionnaire in the QSkin Sun and Health study [22]. The data used in this study were simulated from item difficulties calibrated in a previous study [1]. The Rasch partial credit model [24] was used on the simulated data owing to the different number of categories across items. Further research should focus on whether the psychometric properties of the SC assessment are similar to those of this study if other IRT models are applied.

### Conclusions

The contributions of this study are (1) overcoming the problem of missing responses that limit CAT development when applying the machine learning algorithm, (2) introducing 3 models available on Microsoft Excel and the k-fold cross-validation in Weka software, and (3) demonstrating an app that incorporates Rasch CAT with numerous parameters in LR.

The 30-item SC prediction model, combined with the Rasch web-based CAT, is recommended for classifying SC in adults. An app developed to help patients self-assess SC risk at an early stage is required for application in the future.

## Appendix

Multimedia Appendix 1 Data deposited at OSF (Open Science Framework) research sharing platform.

Multimedia Appendix 2 K-fold cross validation performed in Weka.

Multimedia Appendix 3 Detailed information about the three models used in this study.

## Abbreviations

AUC: area under the curve
CAT: computerized adaptive testing
CNN: convolutional neural network
FN: false negative
FP: false positive
IBk: instance-based learner
IRT: item response theory
KNN: k-nearest neighbors
LR: logistic regression
MM: malignant melanoma
MNSQ: mean square error
NB: naïve Bayes
NMSC: nonmelanoma skin cancer
SC: skin cancer
SC–CAT: skin cancer–computerized adaptive testing
SEM: standard error of measurement
TN: true negative
TP: true positive
