# Supplementary material for: Web-Based Skin Cancer Assessment and Classification Using Machine Learning and Mobile Computerized Adaptive Testing in a Rasch Model: Development Study
Source: JMIR Med Inform. 2022 Mar 9;10(3):e33006. doi: 10.2196/33006 (PMC9282670; doi:10.2196/33006)
Supplement: Multimedia Appendix 2 [file medinform_v10i3e33006_app2.docx]

**Multimedia File 2**

k-fold cross validation performed in Weka at

[https://youtu.be/6ND6HRhQAsY]
