# Supplementary material for: Web-Based Skin Cancer Assessment and Classification Using Machine Learning and Mobile Computerized Adaptive Testing in a Rasch Model: Development Study
Source: JMIR Med Inform. 2022 Mar 9;10(3):e33006. doi: 10.2196/33006 (PMC9282670; doi:10.2196/33006)
Supplement: Multimedia Appendix 3 [file medinform_v10i3e33006_app3.docx]

**The Three models used in this study for more detailed information**

**2.3.1 Naïve Bayes Model or NB Model**

We designed a model under Bayes’ theorem (alternatively Bayes law or Bayes rule) to describe the probability of an event, based on prior knowledge of conditions that might be related to the event [1,2], and for the purpose of classifying the group of melanoma for patients. The equation shown in Eq. (1) is to estimate the cancer probability of (P(A1)) based on condition B(e.g., a specific behavior).

For instance, someone has cancer (denoted by A1 in Eq.(1)) caused by the experience of smoking (B). The probability of cancer caused by the condition B(=P(A1|B)) is determined by the prior probability (=P(A1)), the observed probability(P(B|A1), and the probability in condition B(=P(B)) shown in the denominator in Eq.(1), where P(B) is the summation of all probabilities in behavior B shown in Eq.(2), where A0 denotes the Non-cancer cases and A1 is the cancer cases.

P(A1|B)=$\frac{P(A1\cap B)}{P(B)}$=$\frac{P(B|A1)\times P(A1)}{\sum_{i=0}^{1} P(B|Ai)\times P(Ai)}$, (1)

$P\left( B \right)=P\left( B\cap A1 \right)+P\left( B\cap A2 \right)=\sum_{i=0}^{1} P(B|Ai)\times P(Ai), (2)$

An example for computing the P(A1|B) is illustrated below:

1. The prevalence rate of lung cancer is 2% (=P(A1) in a society.
2. In a survey, the number of smokers in the lung cancer group is 10%(=$P(B|A1))$, and 0.82%($P(B|A0))$ in the Non-cancer group.
3. P(A1|B)=$\frac{P(B|A1)\times P(A1)}{\sum_{i=0}^{1} P(B|Ai)\times P(Ai)}=\frac{0.1\times0.02}{0.0082\times0.98+0.1\times0.02}=0.2$

If no such information on the prevalence rate of lung cancer is provided, P(A1|B) is 0.92(=$\frac{0.1\times0.5}{0.0082\times0.5+0.1\times0.5}$). We can see that the prior probability plays a significant role in Bayes’ theorem.

In the Naïve Bayes (NB) model, the classification of a case with several observed variables is determined via Eq.(3)(or Eqs. (4) and (5) for simplification) as the following steps below:

Class(0 or 1)=$Ｍ\mathrm{ax}$($\prod_{j=1}^{L}\frac{P\left( Bj | A1 \right)\times P\left( A1 \right)}{\sum_{i=0}^{n-1} P\left( Bj | Ai \right)\times P\left( Ai \right)},\prod_{j=1}^{L}\frac{P\left( Bj | A0 \right)\times P\left( A0 \right)}{\sum_{i=0}^{n-1} P\left( Bj | Ai \right)\times P\left( Ai \right)}),$(3)

Class(0 or 1)=$Ｍ\mathrm{ax}\left( \left[ \prod_{j=1}^{L}P\left( Bj | A1 \right) \right]\times P\left( A1 \right),[\prod_{j=1}^{L}P\left( Bj | A0 \right)]\times P\left( A0 \right) \right),$(4)

Class(0 or 1)=$Ｍ\mathrm{ax}\left( \begin{aligned} P\left( B1 | A1 \right)\times P\left( B2 | A1 \right)\times\ldots.. \times P\left( Bn | A1 \right)\times P\left( A1 \right), \\ P\left( B1 | A0 \right)\times P\left( B2 | A01 \right)\times\ldots.. \times P\left( Bn | A0 \right)\times P\left( A0 \right) \end{aligned} \right),$(5)

Step 1: Multiply all proportions across variables and P(A1) in group A1.

Step 2: Multiply all proportions across variables and P(A0) in group A0.

Step 3 or more: If more groups from A0 to An in existence.

Step 4: assign the class number with the maximum value using the maximum likelihood estimation (MLE)[3] in Steps 1 to 3.

Details about the NB model are illustrated in Multimedia File 1 with an MS Excel module.

**2.3.2 The KNN Model**

A KNN model with an MS Excel module is shown in Figure 1. After extracting feature variables, the KNN algorithm was applied through the following steps:

Step 1: Computing the Distance for Each Paired Case(at panel A in Figure 1)

In the n-case training sample, there are n rows and n columns to record the Euclidean distance for each pair case. For instance, the cell D1(=0) is the distance in the first case himself. Cells E1(=9.8) is the distance between the first and the second cases.

Step 2: Sorting the Distances in Columns for Each Case(at the Panel B in Figure 1)

All distances in columns were sorted in ascending order for cases in rows. The shortest distances(=0) are placed in column D, followed by other short distances in the row(e.g., 1.8 and 2.3 in cells E1 and F1 for the first case in row 2).

Step 3: Labeling the Classifications Sorted by Distances in Rows(at the panel C in Figure 1).

All sorted distances in columns were replaced with the corresponding digital labels(e.g., 1 and 0 for SC and Non-SC, respectively). For instance, the cases in the first row are labeled with 0, 0, and 1 in cells D1, E1, and F1.

’
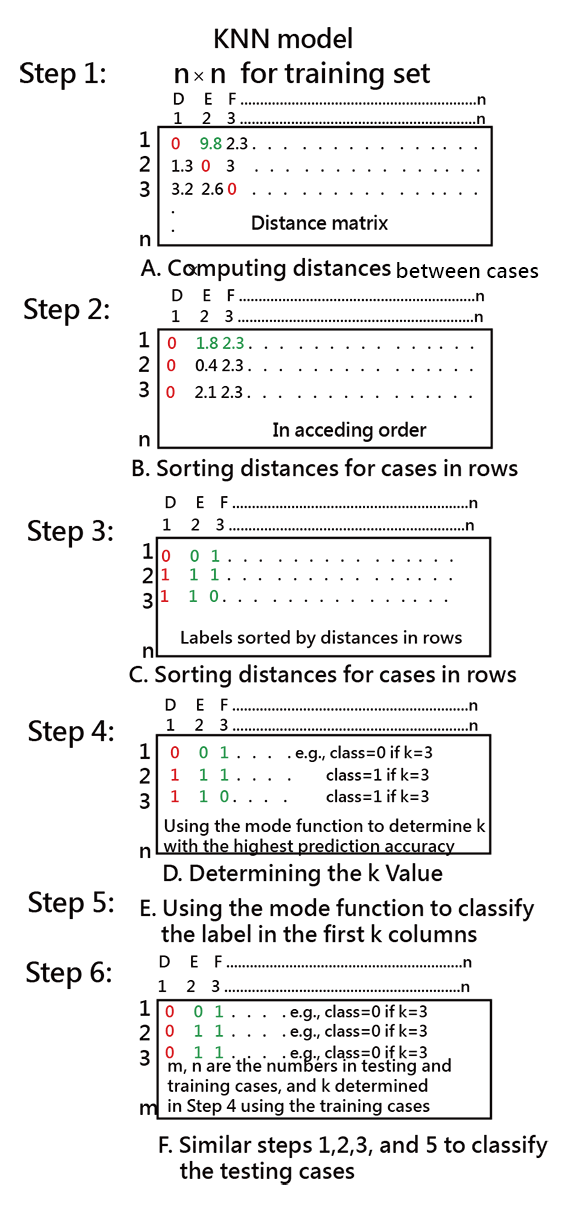


Figure 1 The interpretation of the KNN model

Step 4: Determining the k Values

We simulate the k values(i.e., the number of columns used to predict the classification) from 1 to 10 and select the highest accuracy rate as the nearest k value used for classification.

Step 5: Using the Mode Function in MS Excel to Classify the Case Label in the k

An example of k=3 is selected in step 4, owning to the highest accuracy observed at k=3(i.e., in the first three nearest cases). Before classification for each case in column B (referred to the Excel module in Multimedia File 1), only one class(i.e., labeled as 0 or 1) is possibly predicted. The nearest three distances of matched cases(i.e., columns from D to F in Figure 1, including the first one who is ego-self) are compared using the mode function in MS Excel. For example, the class 1 is assigned into column B(referred to the Excel module in Multimedia File 1) when either one exists in the following instances: {1,1,0}, {1,0,1},and {0,1,1} based on the majority vote of the first k(=3) neighbors in KNN. Otherwise, class 0 is predicted for the case in a row(in Figure 1), accordingly.

Step 6: The k Applied to the Testing Cases

Similarly, the testing cases are compared to the training cases using the k value. The classes in the testing set are then predicted via Steps 1, 2, 3, and 5.

Details about the KNN model are illustrated in Multimedia File 1 with an MS Excel module.

**2.3.3 The Logistic Regression Model or LR Model**

The LR model with an MS Excel module is shown in Figure 2 with the following steps:

Step 1: Actual Labels and Responses for Training Cases

In the n-case training sample, there are classes 0 and 1 in columns and responses, for example, from B to L or more columns(e.g., labels and responses in Figure 2).

Step 2: LR Model Building With L+1 Parameters

The LR model was built. The logit formula (=a+WX) was set for each case. For instance, the formula(=a+b_1_x_1_+b_2_x_2_+b_3_x_3_+…..+b_L_x_L_) is applied to each case in column P. A total of L+1 parameters(i.e., a, b_1_, b_2_, …b_L_) are required to estimate(e.g., putting them in cells from S1 to Z1 in MS Excel).

Step 3: Setting The Probability of Classification

The probability(=prob.=1/(1+exp(-1$\boldsymbol{\times logit))=}\exp\left( \mathbf{logit} \right)\mathbf{/(1+}\exp\left( \mathbf{logit} \right)\mathbf{))}$ was also assigned for each case(e.g., in column R).

Step 4: Setting Predicted Class

The predicted labels were set(i.e., as 0 if prob.<0.5, otherwise as 1).

Step 5: Minimizing the Model Residual

The model residual was determined by the MS function of SUMXMY2(range1:range2), where range1 was composed of the actual labels for each case with two columns(i.e., (0,1) as SC$\mathbf{+}$ and (1,0) as Non-SC$\mathbf{-), and}$range2 was constructed by the corresponding probabilities of SC$\mathbf{+}$ and Non-SC$\mathbf{-.}$ The MS Solver add-in tool was applied to estimate parameters a and W. That is, the intercept coefficients and parameter coefficients were calibrated by the settings in the model optimization process:

Objective: minimizing SUMXMY2(range1:range2)

Parameters: a, b_1_, b_2_, …b_L_

Step 6: The Estimated Parameters Applied to the Testing Cases

The testing cases are set up in Steps 1 to 4. The estimated parameters in the training set are applied to the testing set. The classes in the testing set are then predicted via the parameters of a, b_1_, b_2_, …b_L._

Details about the LR model are illustrated in Multimedia File 1 with an MS Excel module.


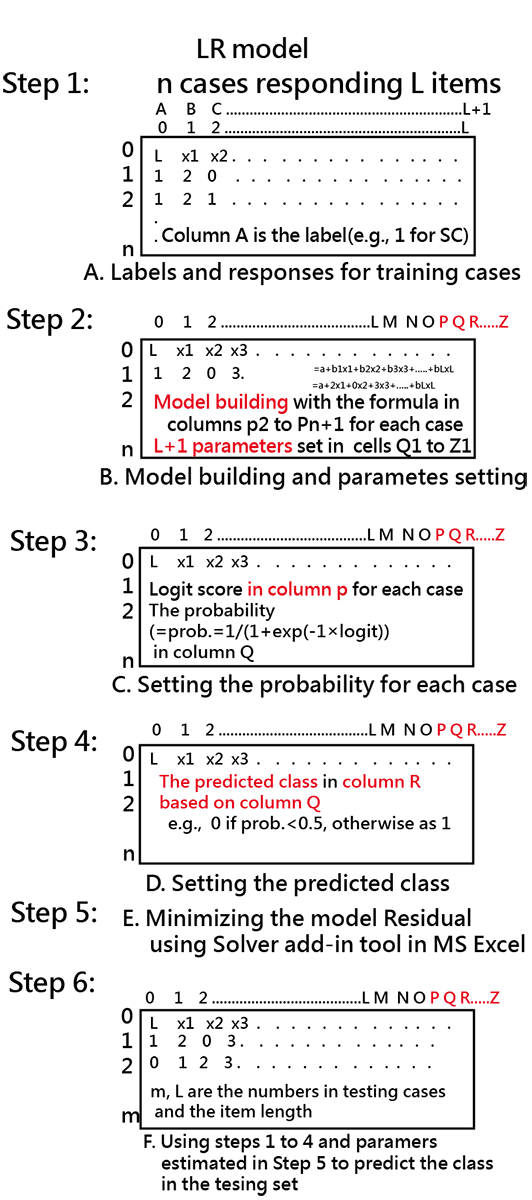


Figure 2 Logistic regression model applied in MS Excel

**References**

1. Joyce J, Zalta EN. Bayes’ Theorem, The Stanford Encyclopedia of Philosophy (Spring 2019 ed.), Metaphysics Research Lab, Stanford University; 2003.
2. Chang CS, Yeh YT, Chien TW, Lin JJ, Cheng BW, Kuo SC. The computation of case fatality rate for novel coronavirus (COVID-19) based on Bayes theorem: An observational study. Medicine (Baltimore). 2020 May 22;99(21):e19925. doi: 10.1097/MD.0000000000019925. PMID: 32481256; PMCID: PMC7249957.
3. Rossi, Richard J. Mathematical Statistics : An Introduction to Likelihood Based Inference. New York: John Wiley & Sons. p. 227, 2018. ISBN 978-1-118-77104-4. Rere LM, Fanany MI,
